# Activation of GPER Induces Differentiation and Inhibition of Coronary Artery Smooth Muscle Cell Proliferation

**DOI:** 10.1371/journal.pone.0064771

**Published:** 2013-06-19

**Authors:** Fen Li, Xuan Yu, Claudia K. Szynkarski, Cong Meng, Beiyan Zhou, Rola Barhoumi, Richard E. White, Cristine L. Heaps, John N. Stallone, Guichun Han

**Affiliations:** 1 Women's Health Division, Michael E. DeBakey Institute, Texas A&M University, College Station, Texas, United States of America; 2 Department of Physiology and Pharmacology, Texas A&M University, College Station, Texas, United States of America; 3 Department of Veterinary Integrative Biosciences, College of Veterinary Medicine and Biomedical Sciences, Texas A&M University, College Station, Texas, United States of America; 4 College of Life Science, Henan Normal University, Xinxiang, Henan Province, P. R. China; 5 Department of Basic Sciences, Philadelphia College of Osteopathic Medicine—Georgia Campus, Suwanee, Georgia, United States of America; Medical University Innsbruck, Austria

## Abstract

**Background:**

Vascular pathology and dysfunction are direct life-threatening outcomes resulting from atherosclerosis or vascular injury, which are primarily attributed to contractile smooth muscle cells (SMCs) dedifferentiation and proliferation by re-entering cell cycle. Increasing evidence suggests potent protective effects of G-protein coupled estrogen receptor 1 (GPER) activation against cardiovascular diseases. However, the mechanism underlying GPER function remains poorly understood, especially if it plays a potential role in modulating coronary artery smooth muscle cells (CASMCs).

**Methodology/Principal Findings:**

The objective of our study was to understand the functional role of GPER in CASMC proliferation and differentiation in coronary arteries using from humans and swine models. We found that the GPER agonist, G-1, inhibited both human and porcine CASMC proliferation in a concentration- (10^−8^ to 10^−5^ M) and time-dependent manner. Flow cytometry revealed that treatment with G-1 significantly decreased the proportion of S-phase and G2/M cells in the growing cell population, suggesting that G-1 inhibits cell proliferation by slowing progression of the cell cycle. Further, G-1-induced cell cycle retardation was associated with decreased expression of cyclin B, up-regulation of cyclin D1, and concomitant induction of p21, and partially mediated by suppressed ERK1/2 and Akt pathways. In addition, G-1 induces SMC differentiation evidenced by increased α-smooth muscle actin (α-actin) and smooth muscle protein 22α (SM22α) protein expressions and inhibits CASMC migration induced by growth medium.

**Conclusion:**

GPER activation inhibits CASMC proliferation by suppressing cell cycle progression via inhibition of ERK1/2 and Akt phosphorylation. GPER may constitute a novel mechanism to suppress intimal migration and/or synthetic phenotype of VSMC.

## Introduction

Vascular smooth muscle cells (VSMCs) constitute the major structural component of the vasculature, and are crucial to maintaining vessel tone, blood pressure, and blood flow. Adult VSMCs retain remarkable plasticity, and can undergo profound and reversible phenotypic changes in response to local environmental stimuli. Normally, VSMCs exhibit a “contractile” or differentiated phenotype characterized by the expression of specific contractile markers (e.g., myosin heavy chain and α-actin) [1]; however, injured VSMCs dedifferentiate and re-enter the cell cycle with an increased rate of proliferation and migration. Further, expression of myosin heavy chain and α-actin is decreased in the proliferative stage. This dedifferentiated phenotype plays a major pathophysiologic role in the development of atherosclerosis, restenosis after angioplasty, and hypertension [2].

Estrogen (17β-estradiol or E2) lowers the risk of cardiovascular disease in women [3], and inhibits VSMC proliferation following injury [4]–[10]. Interestingly, the anti-proliferative action of E2 persists in ER*α*-deficient, ER*β*-deficient, or ER*α*/ER*β*-double-knockout mice [11]–[13]. Thus, the anti-proliferative effect of E2 may involve a novel ER protein. The recently discovered G protein-coupled estrogen receptor 1 (GPER) is a seven transmembrane-domain G protein receptor structurally unrelated to ERα or ERβ, binds E2 with high affinity, and mediates estrogenic signaling [14], [15]. The selective GPER agonist, G-1, lowers blood pressure in either normotensive [16] or mRen2. Lewis hypertensive rats [17], whereas GPER gene knockout female mice exhibit increased blood pressure–presumably due to increased total vascular resistance associated with arterial wall remodeling [18]. In addition, G-1 improves functional recovery from myocardial ischemia-reperfusion by reducing post-ischemic contractile dysfunction and infarct size [19]. Thus, GPER is a potential mediator of estrogen action on coronary arteries, but whether GPER plays a role in coronary artery smooth muscle cell (CASMC) proliferation is unknown.

In the present study, we demonstrate that G-1 inhibits serum-induced VSM proliferation in both human and porcine coronary arteries, and have characterized downstream signaling events. We have also investigated the effect of G-1 on the re-differentiation and migration of CASMC. Our data provide new insight into the mechanisms of estrogen receptor signaling in VSMC, and reveal GPER to be a target for the development of therapeutic strategies in vascular diseases.

## Materials and Methods

### Reagents

Recombinant human PDGF-BB was purchased from Sigma Company. G-1 was purchased from Cayman Chemical Company and dissolved in DMSO, and the concentration of DMSO was less than 0.05% in the control and drug-containing medium.

### Isolation of porcine CASMCs and culture of porcine and human CASMCs

Porcine hearts were obtained from and permitted by local abattoirs, K&C meat processing. Coronary arteries were dissected, and CASMCs were enzymatically dispersed [20]. Primary cultured porcine CASMCs (PCASMC) and human CASMCs (HCASMC, Cascade Biologics, C-017-5C) were maintained in Medium 231 with Smooth Muscle growth Supplement (SMGS) (GIBCO USA), 100 µg/ml penicillin and 100 µg/ml streptomycin. Cell cultures were kept at 37°C and under 5% CO_2_ in a humidified incubator. CASMCs were cultured to 80% confluence, and we employed passage 2–3 for porcine CASMCs or passage 6–8 for human CASMCs.

### Cell proliferation assay

CASMCs were seeded onto 6-well plates (1×10^5^ cells per well) for 24 h for attaching and then serum-deprived in phenol red free MEMα medium (GIBCO USA) for 72 h before treatment with G-1 or PDGF-BB (10 ng/ml) with 10% Charcoal Stripped FBS (FCS) (GIBCO USA). Cell proliferation was determined by daily counting the number of cells in triplicate.

### Cell cycle progression analysis

Cell cycle progression assay was performed by RNase staining followed by FACS (fluorescence-activated cell sorter) analysis. Distribution of CASMCs cells in the cell cycle was determined by flow cytometry of propidium iodide-stained nuclei as described by Odenlund et al [21]. Briefly, flow-cytometric DNA analysis was performed in a FACS Calibur flow cytometer equipped with data acquisition capability.

### Cellular migration analysis

Analysis of cellular motility/migration was carried out using Culture-Inserts ready to use in a μ-Dish 35 mm (ibiTreat, item #: 81176, IBIDI), which allows performing high resolution microscopy in a 35 mm Petri–dish with 12 mm walls. PCASMCs were seeded in Medium 231 with SMGS (GIBCO USA), 100 µg/ml penicillin and 100 µg/ml streptomycin. When cells reached 100% confluence, the inserts were taken out and a 500 µM gap was left in each dish. Cells were then grown in fresh control media, or media+drug treatment (e.g., G-1 or G-1+G-15), and cells were allowed to migrate for 48 h. Images were collected with a Stallion Digital Imaging workstation (Carl Zeiss) equipped with a HQ CoolSnap camera (Photometrics) and a 5× objective. Five images per treatment were collected before and after 48 h following removal of the inserts. Within a specific image five different distances were measured from the edge marked by a dotted black line. Data collected represent the mean distance traveled from the edge.

### Western blot analysis

After human CASMCs (1×10^5^) were cultured in a 6 cm diameter dish for 48 h, they were starved in serum-free medium for another 48 h. Cells were then treated with G-1 (1 µM, from Calbiochem) and PDGF-BB (10 ng/ml, from Sigma-Aldrich Corporation). For cell cycle protein extracts, cells were starved in serum-free medium for 72 h before G-1 treatment. Harvested cells were disrupted, and the protein concentration was determined using BCA assay according to the manufacturer's instructions. Proteins were detected with the following antibodies: anti-cyclinB1 (sc-7393 Santa Cruz, 1∶200), anti-cyclin D1 (sc-20044 Santa Cruz, 1∶200), anti-p21 (sc-6246 Santa Cruz, 1∶100), anti-pERK1/2 (No. 9101s Cell Signaling, 1∶1000), anti-pAKT (No. 4060s Cell Signaling, 1∶1000), anti-α-actin (ab5694 Abcam, 1∶4000), anti-SM22α (ab14106 Abcam, 1∶5000), anti-β-actin (ab8227 Abcam, 1∶1000), or anti-GAPDH (sc-25778 Santa Cruz, 1∶1000). After chemiluminescence detection (EMD Millipore, Billerica, MA, USA), Image J software was used for data analysis. The experiments were replicated three times.

### Immunocytochemistry

Human and porcine CASMCs seeded on 12 mm glass coverslips placed in 6 well plates were serum-deprived for 48 h, and then treated for 1–2 days with vehicle, SMDS, or G-1in phenol red free MEMα medium. Cells were then fixed in 10% buffered formalin for 10 min, washed twice with PBS, and permeabilized in 0.2% Triton X-100 and PBS for 10 min. CASMCs were washed twice with PBS, and incubated with 4% BSA-PBS for 1 h, then with anti-α-actin antibody overnight at 4°C and incubated with FITC-conjugated anti-rabbit IgG antibody (PA1-29388 Thermo Scientific Pierce) for 1 h at room temperature in darkness. After three washes with PBS, the coverslips were mounted for imaging.

### Transfection of siRNA

Human CASMCs were transfected in 6- or 12-well dishes at 30–60% confluence with 75 nM siRNA using Lipofectamine 2000 (Invitrogen) according to the manufacturer's instructions. The cells were studied 48 h after transfection. Transfected cells were then treated with G-1(10 nM or 1 µM) for 48 h. For determination the effect of silencing GPER on decreased PCNA expression and cell morphology change caused by G-1 treatment, immunocytochemistry was carried out as described above. For determination of silencing efficiency, Western blotting was performed and membrane was probed with GPER antibody.

### Statistics analysis

Data are presented as means ± standard deviation (SD) and analyzed with Prism program (GraphPad Software Inc., San Diego, CA). One-way or 2-way analysis of variance (ANOVA) followed by Tukey's multiple comparison test paired with repeated measures were carried out for statistical analysis as appropriate. P values less than 0.05 were regarded as statistically significant.

## Results

### GPER activation reduces CASMC proliferation

**Figure 1:**
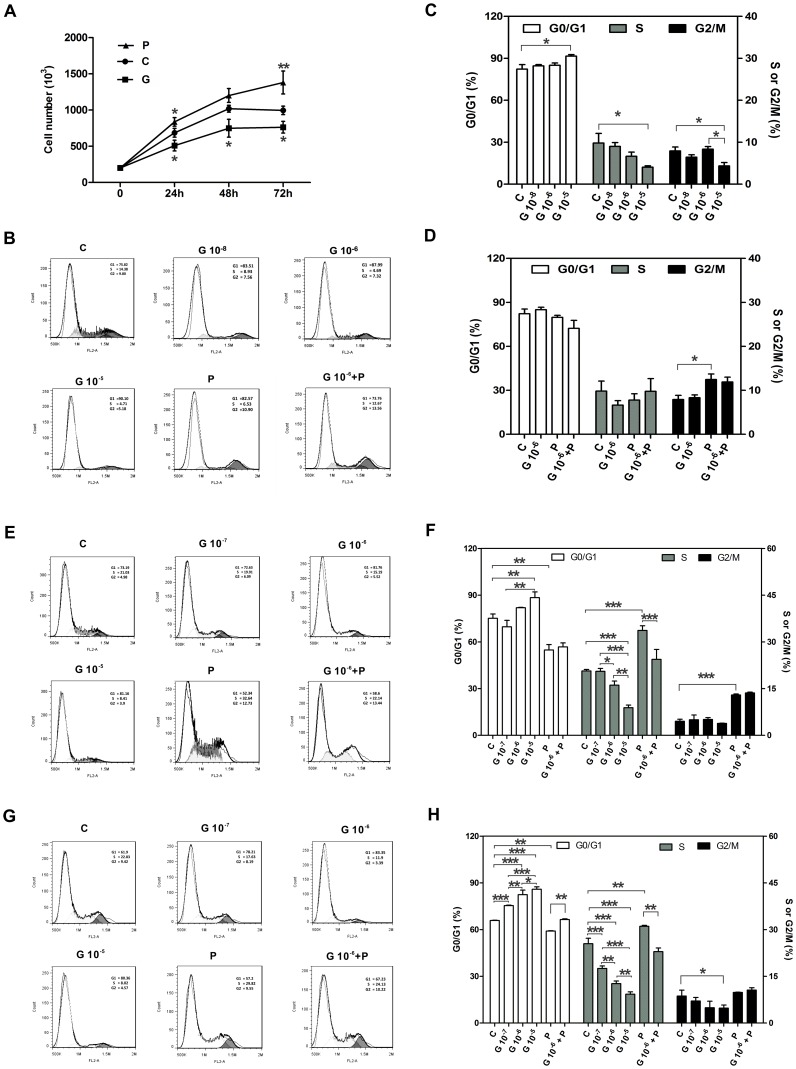
Reduced proliferation and delayed cell cycle progression of G-1 treated CASMCs cells. A: Cell proliferation curves of G-1 and/or PDGF-BB treated HCASMC cells. CASMCs were synchronized by 3 days of culturing in serum free and phenol red-free DMEM medium. Then 10% fetal calf serum (FCS) was added into medium and cells were counted manually with trypan-blue method (trypan-blue method of exclusion). B, E and G: HCASMC (B) and PCASMC (E, G) cell cycle distribution was determined by propidium iodide staining of DNA content and flow cytometry. After synchronized, 10% FCS was added and cells were treated with vehicle as control (C); G-1 (G, 10^−8^–10^−5^ M); 10 ng/ml PDGF-BB (P) or 1 µM G-1 plus 10 ng/ml PDGF-BB (G+P) at 24 or 48 hours. Twenty-thousand cells per sample and three replicates per group were collected. Representative histograms are shown in C (G-1 concentration response); D, G+P, 1 µM G-1 plus 10 ng/ml PDGF-BB, for 48 h treatment of HCASMC; F, 24 hour treatment and H, 48 hour treatment of PCASMC with 1 µM G-1 plus 10 ng/ml PDGF-BB. Representative histograms are shown as the mean ±SD (n = 3), a significant difference is indicated by either *p<0.05; ** p<0.01 or ***p<0.001, one-way or two-way ANOVA.

We examined the action of GPER in controlling both primary human and porcine CASMC proliferation. Human CASMCs (passage 6–8) treated with G-1 displayed reduced cell growth (Figure 1A), for example, treating cells with 1 µM G-1 for 24 h reduced cell growth by 26%. In contrast, the pro-proliferative agent platelet-derived growth factor (PDGF-BB, 10 ng/ml, 24 h treatment) increased cell number by 22%. These results indicate that activation of GPER by G-1 represses cell proliferation of human CASMCs.

The suppressive effect of G-1 on CASMCs was further investigated by analyzing cell cycle progression (Figure 1; table 1–3). Cells were cultured in 10% FCS after synchronization. G-1 (10^−5^ M) significantly decreased the proportion of human CASMCs in phases S and G2/M by 58.76% and 44.75% respectively after 48 h treatment (G-1 vs control, P<0.05, n = 3; Fig. 1B, 1C). The addition of 10 ng/ml PDGF-BB in 10% FCS medium clearly stimulated cell growth with a marked increase in the cell proportion of S phase and G2/M cells at 48 h (Figure 1B, 1D; table 1). Although data indicated a clear trend for 1 µM G-1 48 h treatment lowering the proportion of stimulated cells in G2/M phase, this effect did not reach statistical significance compared to controls (Figure 1B, 1D). The relative higher passage of the human CASMCs might have contributed to the lesser effectiveness of G-1 on the addition of PDGF-BB in 10% FCS compared to 10% FCS alone stimulated cell growth.

**Table:** **Table 1.** The effect of G-1 treatment on human CASMCs for 48 hours.

	G0/G1		S		G2/M	
	Mean 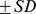	Change after treatment (%)	Mean 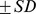	Change after treatment (%)	Mean 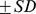	Change after treatment (%)
Control	82.27 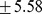		9.82 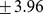		7.91 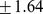	
G 10^−8^	84.58 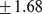	2.81	9.00 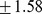	−8.35	6.42 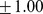	−18.84
G 10^−6^	85.03 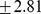	3.26	6.64 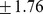	−32.38	8.33 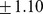	5.31
G 10^−5^	91.58 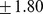	10.95	4.05 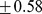	−58.76	4.37 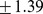	−44.75
PDGF	79.77 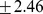	−2.96	7.78 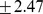	−20.77	12.45 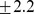	57.40
G10^−6^+P	72.27 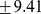	−12.54	9.78 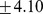	−0.41	11.90 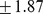	50.44

**Table:** **Table 2.** The effect of G-1 on porcine CASMCs after 24 hour treatment.

24 h	G0/G1		S		G2/M	
	Mean 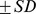	Change after treatment (%)	Mean 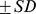	Change after treatment (%)	Mean 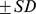	Change after treatment (%)
C	75.15 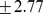		20.68 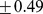		4.51 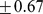	
G 10^−7^	69.68 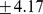	−7.28	20.56 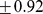	−0.58	5.01 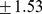	11.09
G 10^−6^	81.93 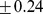	9.02	16.12 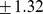	−22.05	5.09 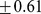	12.86
G 10^−5^	88.43 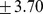	17.67	8.88 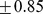	−57.06	3.84 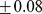	−14.86
P	54.80 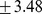	−27.08	33.69 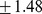	62.91	12.96 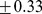	187.36
G10^−6^+P	56.76 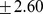	−24.47	24.40 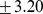	17.99	13.63 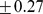	202.22

**Table:** **Table 3. **The effect of G-1 on porcine CASMCs after 48 hour treatment.

48 h	G0/G1		S		G2/M	
	Mean 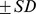	Change after treatment (%)	Mean 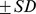	Change after treatment (%)	Mean 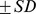	Change after treatment (%)
C	65.86 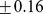		25.48 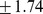		8.66 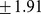	
G 10^−7^	75.40 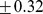	14.49	17.53 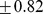	−31.20	7.07 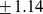	−18.36
G 10^−6^	82.42 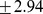	25.14	12.66 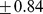	−50.31	4.92 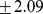	−43.19
G 10^−5^	85.94 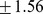	30.49	9.27 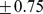	−63.62	4.79 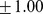	−44.69
P	59.10 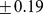	−10.26	31.08 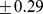	21.98	9.82 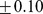	13.39
G10^−6^+P	66.48 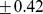	0.94	22.93 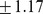	−10.01	10.59 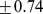	22.29

The numbers in Tables 1–3 are from Figure 1 B–H.

Swine models of coronary dysfunction are similar to those of humans in response to injury or pathophysiological conditions [22]. Therefore, we also employed primary porcine CASMCs and tested for GPER activation at a low passage (p2) (Figure 1E–1H; table 2–3). Compared to human CASMCs, the anti-proliferative effect of G-1 on porcine CASMC was more robust. Treatment with G-1 (10^−7^, 10^−6^, or 10^−5^ M) alone significantly decreased the proportion of S phase cells at 24 h and G2/M-phase cells at 48 h, while increased accumulation of cells at G0/G1 phase. Furthermore, G-1 significantly suppressed 10% FCS plus 10 ng/ml PDGF-BB-induced cell proliferation, as evidenced by a marked decrease of cell population in S phase (p<0.01, n = 4). These data indicate that G-1 significantly inhibits the proliferation of porcine CASMCs by slowing down the progression of the cell growth cycle from G0/G1 to S and G2/M-phase. Together with the data from human CASMCs, these data suggest that G-1-activated GPER can inhibit proliferation by controlling cell cycle progression in CASMCs in a concentration- and time-dependent manner.

### GPER activation represses cell cycle progression by inducing expression of p21 in human and porcine CASMCs

**Figure 2:**
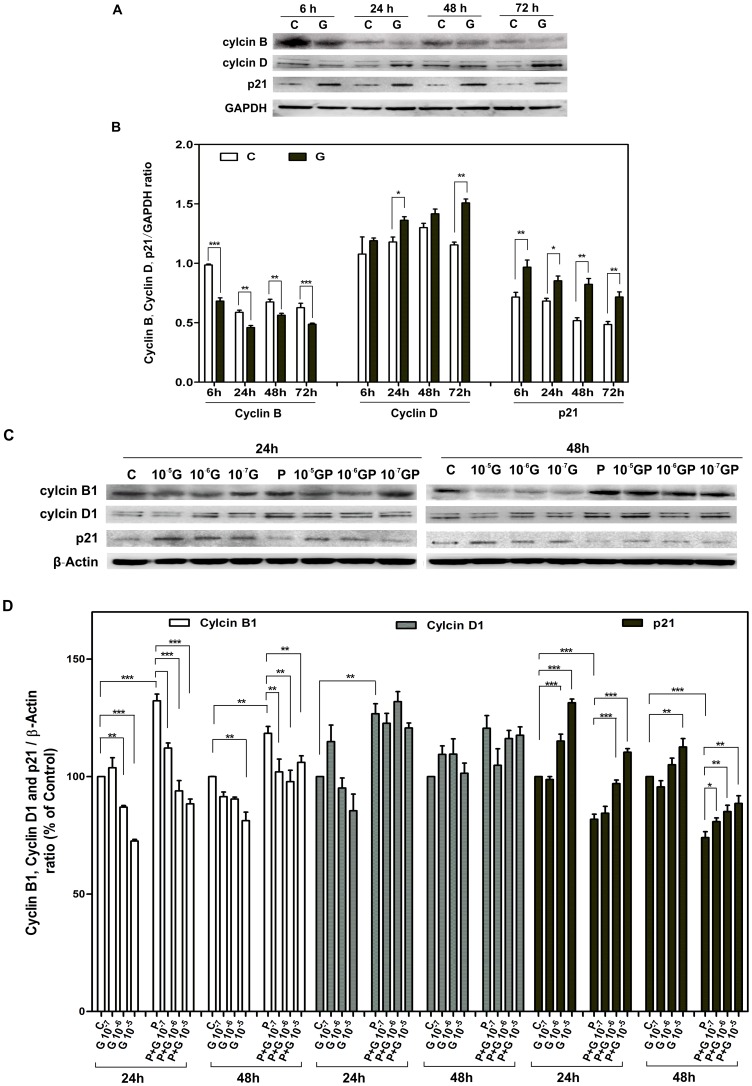
Effects of G-1 treatment on the protein level of cyclinB1, cyclinD1 and p21 in CASMCs. A and C: Western blot results of cyclinB1, cyclinD1 and p21 protein levels in human CASMCs (A) and porcine CASMCs (C). CASMCs were synchronized by 3 days of cultivation in serum-free and phenol red free medium followed by vehicle treatment as control (C); G-1(G, 10^−7^–10^−5^ M); 10 ng/ml PDGF-BB (P); and G-1(10^−7^–10^−5^ M) plus 10 ng/ml PDGF treatment (GP or P+G) for 24 or 48 hours in the presence 10% FCS. Total cell extracts (1×10^6^) were subjected to Western-blot analyses for cyclinB1, cyclinD1 and p21 level. B and D: Quantitative densitometric analyses of band intensities from 3 independent experiments. Data are normalized by GAPDH or β-Actin, expressed as the mean±SD (n = 3). A significant difference is indicated by either *(p<0.05); **(p<0.01) or *** (p<0.001) (one-way ANOVA). Representative histograms are shown.

There is increasing evidence for a critical role of the cyclin-dependent kinase inhibitor (CDK-I) p21 in repressing VSMC growth [23], [24]. Accordingly, we examined whether p21 and other cell cycle regulatory molecules (i.e., the G1-phase–specific cyclin D1, and the G2/M-phase–specific cyclin B1) are involved in anti-proliferative effects of GPER. Human CASMCs growth was synchronized by 3-day serum deprivation, and then cells were treated with 1 µM G-1 in 10% FCS. Cells were collected at 6, 24, 48, or 72 hours for immunoblot analysis (Figure 2A and 2B). Immunoblot studies revealed that p21 proteins were up-regulated in G-1-treated cells at all-time points compared to vehicle-treated cells (Figure 2B). Unexpectedly, G-1 treatment increased cyclin D1 level (Figure 2B), but almost completely prevented cyclin B1 accumulation (Figure 2B). These data indicate that GPER activation by G-1 treatment accumulates cell population in G1 phase, but hinders the cell cycle from entering G2/M phase.

We then tested these effects of G-1 on porcine CASMCs. The protein levels of cell cycle regulatory molecules were examined at 24 and 48 hours by immunoblot analysis (Figure 2C and 2D). As expected, PDGF-BB clearly up-regulated expression of both cyclins D1 and B1, while p21 expression was down-regulated compared to controls (Figure 2D), further confirming the cell cycle accelerating effect of PDGF-BB in CASMCs. G-1, on the other hand, not only increased p21 expression and nearly prevented cyclin B1 accumulation at both 24 and 48 hours (in the presence of 10% FCS), but also inhibited the effects of PDGF-BB on p21 and cyclin B1 in a similar concentration-dependent manner (Figure 2D). However, the effect of G-1 on PDGF-BB-induced cyclin D expression was not significant (Figure 2D). Taken together, our results demonstrate that G-1 blocks cell cycle progression in late G1 phase before cyclin D1 degradation occurs and before cyclin B1 accumulates.

### GPER activation decreases phosphorylation of AKT and ERK1/2

**Figure 3:**
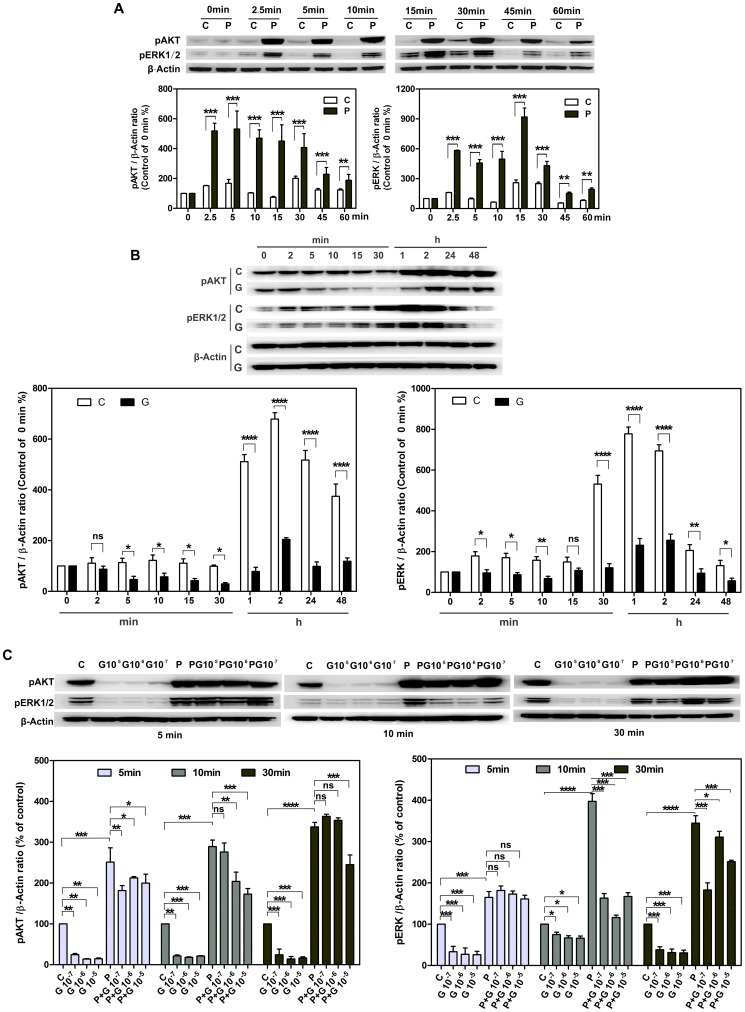
G-1 inhibits the phosphorylation of Akt and ERK1/2 in CASMCs. A and B: HCASMCs were cultured in serum and phenol red free medium for two days followed by PDGF-BB (10 ng/ml) (A) or G-1 (1 µM) treatment (B) for indicated time in the presence of 10% FCS. C: PCASMCs were cultured in the same medium for two days followed by G-1(10^−7^–10^−5^M); PDGF-BB (10 ng/ml); and G-1(10^−7^–10^−5^M) plus PDGF-BB (10 ng/ml) treatments for indicated time in the presence of 10% FCS. Total cell extracts (1×10^6^) were subjected to Western-blot analyses for Phospho-Akt and ERK1/2 level. Under the Western blot panels, a quantitative representation of the expression analysis from 3 independent experiments is shown. Vehicle-treated CASMCs cells were used as control. Data are normalized by β-Actin and expressed as means ±SD (n = 3). A significant difference is indicated by either **** p<0.0001, *** p<0.001, ** p<0.01 or * p<0.05(one or two-way ANOVA); C, G and P represent control, G-1 and PDGF-BB treatment sample respectively, ns indicates no significant difference.

To understand the pathways that mediate GPER activation regulated cell proliferation, we next examined the effects of G-1 on extracellular signal-regulated protein kinases (ERKs)-1 and 2 (ERK1/2) and Akt – two signaling systems that powerfully impact cell proliferation in breast and prostate cancer cells [25]–[27]. After serum deprivation, human CASMCs (passage 7–8) were treated with 10 ng/ml PDGF-BB for the indicated times (total period of 60 minutes) in the presence of 10% FCS. Control groups were treated with vehicle only. PDGF-BB-treated cells showed a significant increase of phosphorylated Akt and ERK1/2 over 60 min compared to controls (Figure 3A; P<0.05, n = 3). In contrast, human CASMCs treated with 1 µM G-1 exhibited decreased phosphorylation of both ERK1/2 and Akt at each time point, with the exception of p-Akt at 2-minutes, compared to vehicle-treated controls (Figure 3B).

Because our cell cycle results revealed a stronger G-1 effect in porcine CASMCs (passage 2) compared to human CASMCs (passage 7–8), we next tested the effect of G-1 on ERK1/2 and Akt phosphorylation induced by 10% FCS or 10% FSC plus 10 ng/ml PDGF-BB in porcine CASMCs. G-1 (10^−7^–10^−5^ M) inhibited FCS-induced phosphorylation of ERK1/2 and Akt in a concentration-dependent fashion. Moreover, G-1 inhibited the enhanced phosphorylation of Akt and ERK induced by the addition of 10 ng/ml PDGF-BB in 10% FSC medium (Figure 3C). These results demonstrated that the inhibitory effects of GPER activation by G-1 in CASMCs are mediated by suppressing ERK1/2 and Akt activation.

### G-1 stimulation changes CASMC morphology by increasing the protein level of α-Actin and SM22

**Figure 4:**
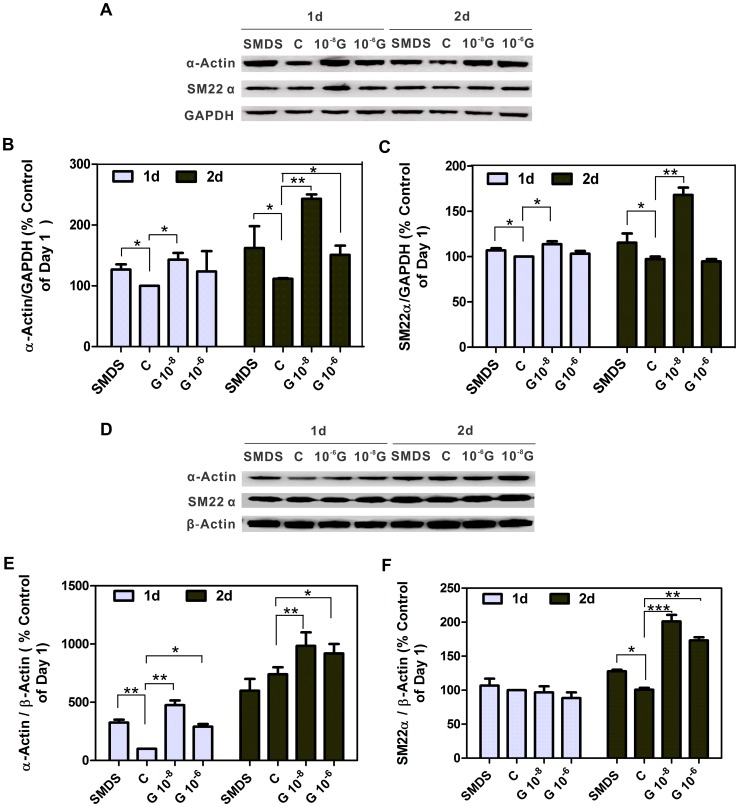
Effect of G-1 treatment on cell morphology in cultured CASMCs. Human and porcine CASMCs were incubated in the presence of either a SMDS or G-1 (1 µM and 10 nM) for 1 and 2 day followed by immunostaining using anti-α-smooth muscle antibody and FITC-conjugated anti-rabbit IgG secondary antibody (green).

**Figure 5:**
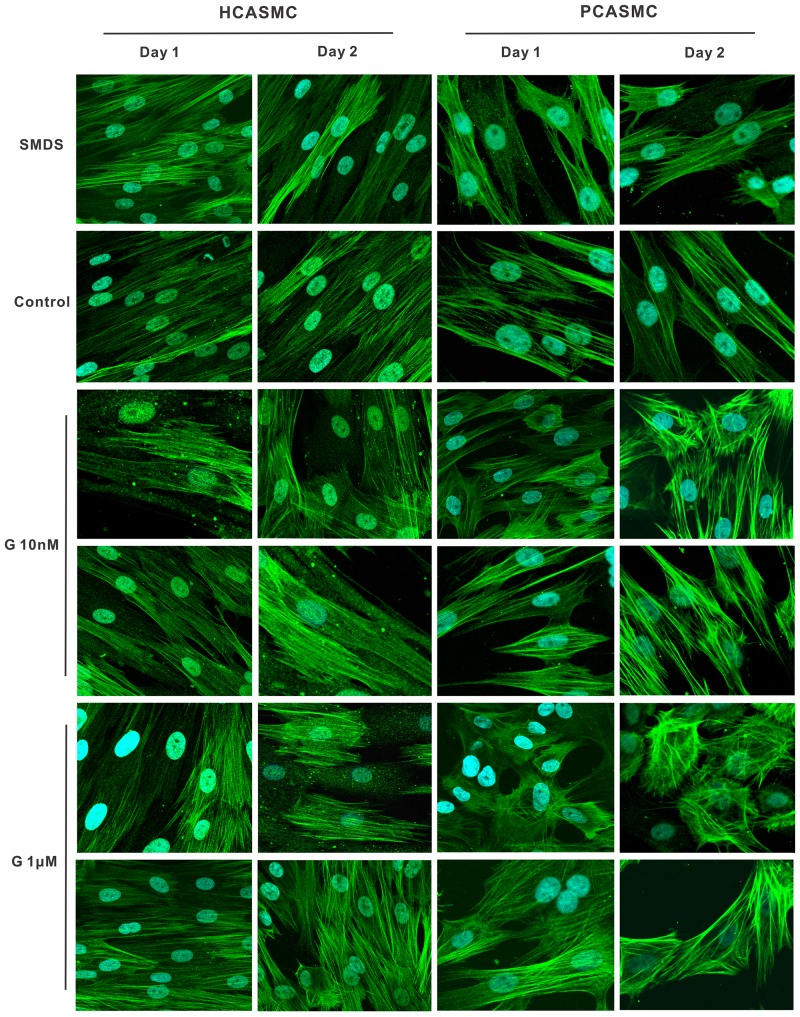
Effect of G-1 treatment on the protein level of α-smooth muscle actin and SM22α in CASMCs. A and D: changes of α-smooth muscle actin and SM22α protein in SMDS and G-1 treated human (A) and porcine (D) CASMC. Expression of α-smooth muscle actin and SM22α protein were determined by immunoblot analysis. SM22α and α-smooth muscle actin were increased in G-1 treated cells in a concentration and time-dependent manner. SM22α and α-smooth muscle actin expression was quantified by densitometric analysis from 3 independent experiments. Data are normalized by GAPDH (HCASMC) and β-Actin (PCASMC), expressed as the mean ±SD (n = 3). *P<0.05, **P<0.01 vs control (one-way ANOVA). Representative histograms are shown in B, C and E, F.

Several lines of evidence suggest that p21 up-regulation and cell cycle arrest are necessary for cell differentiation [28], [29]. Therefore, we postulated that G-1 might contribute to SMC differentiation. To test this possibility, we used Smooth Muscle Differentiation Supplement (SMDS) to induce differentiation of normal human and porcine CASMCs [30]. After 3-day synchronization, 10% FCS was added to the medium and cells were then treated with 10 nM or 1 µM G-1. Vehicle-treated SMCs exhibited a typical SMC morphology: flattened and spindle shaped with central oval nuclei and long cytoplasmic extensions. Confluent cells appear aligned in parallel so that the broad nuclear region of a cell lies adjacent to the thin cytoplasmic area of another forming a “hill-and-valley” appearance. Cells treated with SMDS displayed a change in cellular morphology from slender stellate cells to enlarged rectangular-shaped cells. Furthermore, immunocytochemistry demonstrated increased amounts of smooth muscle α-actin. A more contractile phenotype and increased α-actin was seen in both G-1-treated groups. Cells showed more enlarged rectangular or even triangular shaped cells in human CASMCs (Figure 4, left side two panels). These phenotypic characteristics were even more pronounced in porcine CASMCs (Figure 4, right side two panels). After prolonged G-1 exposure some cells exhibited an increased stacking of α-actin fibers giving the appearance of a “bird's nest” shape, and the upper panels of G-1 (10 nM and 1 µM)-treated human and porcine CASMCs were selected areas for this “bird's nest” shape morphology change. Further, G-1 produced a much greater increase of smooth muscle α-actin and SM22 α than that of cells treated with SMDS alone (Figure 5). For example, α-actin expression induced by 10 nM G-1 (2-day treatment, human and porcine CASMCs) was 46.62% and 79.23% greater, respectively, compared to α-actin expression in SMDS-treated cells P<0.01 (n = 3). Collectively, these data demonstrate that GPER activation induces a differentiated VSMC phenotype.

### GPER activation inhibits PCNA expression in CASMCs

**Figure 6:**
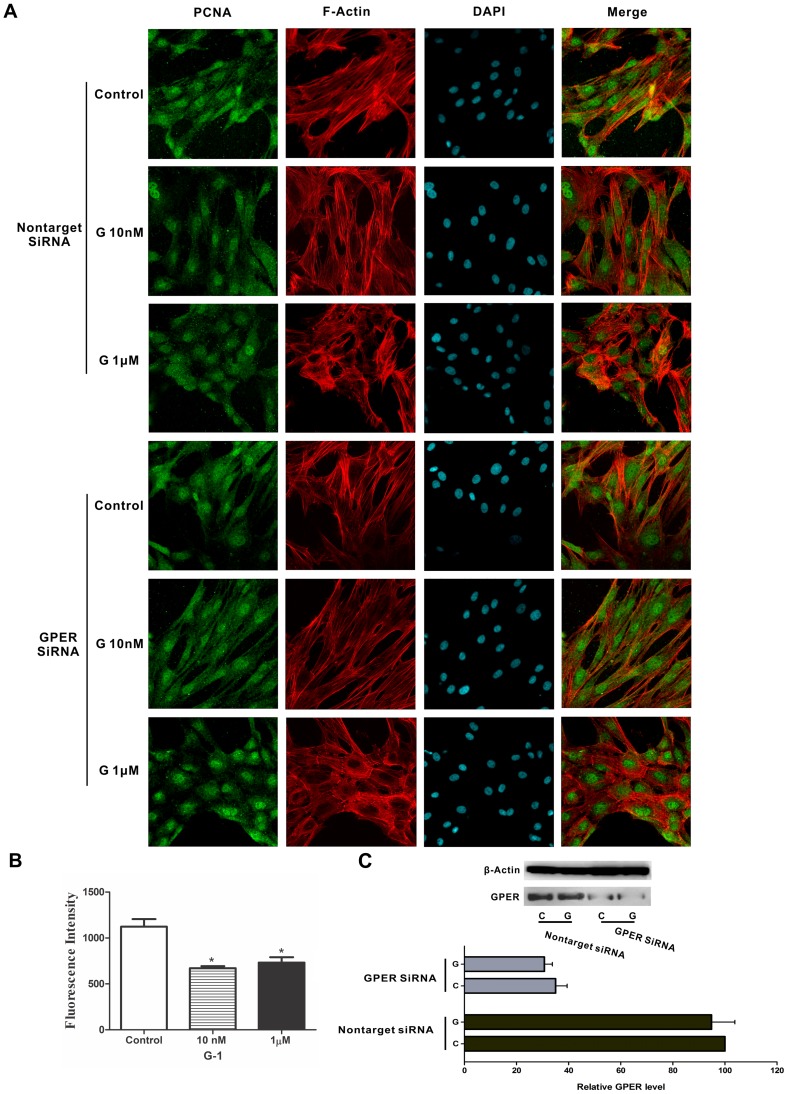
Down-regulation of GPER reverses the decreased expression of PCNA and cell morphology changes caused by G-1. (**A**) HCASMC cells transfected with non-targeted siRNA or GPER siRNA were incubated with G-1 (1 µM or 10 nM) for 48 h, followed by immunostaining using anti-PCNA antibody and FITC-conjugated anti-rabbit IgG secondary antibody (green). Cells were then stained by acti-stainTM555 Fluorescent phalloidin for F-actin to reveal cell morphology. Slides were mounted with ProLong Gold anti-fade regent with DAPI (Invitrogen Life Technologies, Gaithersburg, MD, USA) for imaging. (B) The expression of PCNA were presented as mean fluorescence intensities ± S.E. of at least 50 cells collected from at least 5 random areas per sample and were analyzed statistically by ANOVA followed by Tukey's multiple comparison test. *Indicates significant difference from control at p<0.05. (C) GPER protein levels were measured by immunoblotting in siRNA-transfected cells 48 h following transfection.

Proliferating cell nuclear antigen (PCNA) expression increases as mitogens (such as PDGF) or serum stimulates VSMC proliferation [31]. In order to determine the effect and specificity of G-1 on proliferation we knocked-down expression of GPER via siRNA transfection studies, and then determined the effect of G-1 on PCNA expression and cell morphology in serum-stimulated human CASMCs. In control cells transfected with non-target siRNA, 10 nM or 1 µM G-1 clearly decreased serum-induced increases in PCNA expression, (Figure 6 A, B) and also changed cell morphology into a more enlarged rectangular shape. In contrast, G-1 failed to exert an inhibitory effect on PCNA expression in cells transfected with GPER siRNA, and the effect on cell morphology was less compared to controls (Figure 6A). Western blotting confirmed the efficiency of GPER silencing with siRNA, as GPER expression deceased by 65–69.3% (Figure 6C). Collectively, these results strongly suggest that G-1 inhibits CASMC proliferation via a GPER-mediated mechanism.

### GPER activation inhibits CASMC migration

**Figure 7:**
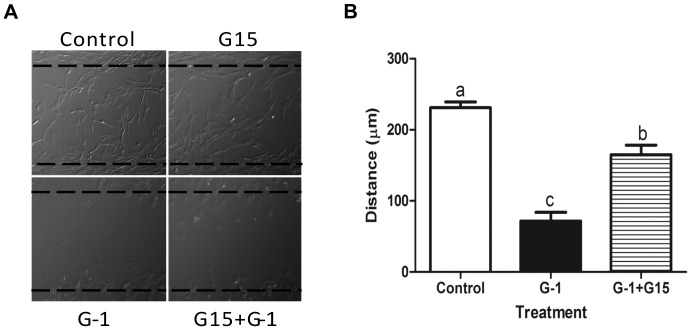
Effect of GPER stimulation on CASMC migration. (A) Representative of three experiments of porcine CASMC migration study. Cellular migration was assessed by using scratch-wound assay. Cells were cultured in ibidi μ-dish culture insert until they reached confluence. Inserts (500 µm; dashed lines) were then removed, and cells were exposed to control media or media supplemented with either 1 µM G-1 or 1 µM G-1+5 µM G15. Five images per treatment were collected immediately and then 48 h following insert removal. Within a specific image five different distances were measured from the edge of the dashed-line. (B) Each bar represents the mean distance traveled from the edge +/− SEM. Comparison between the different treatments was done with One-way ANOVA followed by Tukey's multiple comparison test, and treatment groups identified by different letter (a,b, or c) indicate significant difference (p<0.05) between each of the three experimental groups.

Vascular smooth muscle cell migration accompanies proliferation, and plays important role in the pathogenesis of atherosclerosis or arterial injury. Therefore, we tested the effect of G-1 on migration of porcine CASMCs. Cells were seeded on 35 mm dishes with Culture-Inserts, and grown in Medium 231 with SMGS. When cells reached 100% confluence, the inserts were removed leaving a 500 µM gap in the growing cell population. Fresh growth medium was then added to the cells, which were exposed to different treatment conditions (Figure 7). After a 48 h incubation, cells from the control group migrated to the center of the dish, but did not fully cover the gap. 1 µM G-1, however, completely inhibited cellular migration stimulated by the growth medium (p<0.05; Figure 7B). Addition of 5 µM G15 to the growth medium did not affect control cell migration; however, the inhibitory effect of G-1 on cellular migration was attenuated significantly by G15 (p<0.05). Collectively these results suggest that GPER activation inhibits CASMC migration.

## Discussion

GPER is a G protein-coupled receptor functioning independently from ERα and ERβ to regulate cellular and physiological responsiveness to estrogen [14], [30]. To our knowledge, this study is the first to demonstrate effects of a selective GPER agonist, G-1, on coronary artery SMC differentiation, proliferation and migration, and to propose key mediators of this response. Previous studies indicate that GPER activation promotes cancer cell proliferation and migration primarily by producing connective tissue growth factor in a human breast cancer cell line SKBr3 [32], [33]. In addition, tamoxifen, an ERα antagonist but GPER agonist, induces abnormal endometrial thickening and cell proliferation [34], [35]. Expression of both GPER and ERα along with active EGFR signaling, is required for E2-stimulated and G-1–stimulated proliferation of ovarian cancer cells [36]. In contrast to these proliferative effects, activation of GPER by G-1 inhibits growth of androgen-dependent and -independent prostate cancer cells *in vitro* and PC-3 xenografts *in vivo* [27]. Thus, GPER functions in a tissue- or cell-specific manner. In blood vessels the VSM layer is enlarged in arteries from GPER gene knockout female mice, indicating that GPER helps maintain VSMCs in a dedifferentiated state [18]. In addition, G-1 reduces serum-stimulated human umbilical VSMC proliferation [16]. These findings are consistent with our results of G-1 inhibition of CASMCs proliferation or migration. Involvement of GPER in these inhibitory responses to G-1 was substantiated by our experiments indicating that the effect of G-1 on proliferation was attenuated in cells expressing GPER siRNA, whereas G-1-induced inhibition of migration was inhibited significantly by G-15, a selective GPER antagonist.

E2 inhibits serum-stimulated cell growth of human CASMCs by 57% and arrests PDGF-BB stimulated cell cycle at the G1 phase in human aortic VSMC [10]. In contrast, we found that G-1 failed to effectively repress cell cycle progression after 24 hours in human CASMCs (passage 7–8). However, G-1 (24 hours) effectively inhibited serum-induced growth of primary CASMC from pig coronary artery, but failed to inhibit serum plus PDGF-induced cell growth of these cells. These findings indicate that the anti-proliferative effect of G-1 on VSM is distinct from that of E2, and suggest a stronger anti-proliferative effect compared to G-1. Nonetheless, Haas et al. [16] found that G-1 reduced proliferation of human umbilical vein SMCs by 60–80%. Interestingly, these cells lose expression of ERα and ERβ in culture, yet retain full GPER expression. Therefore, it appears that activation of GPER, like nuclear ER, exerts an anti-proliferative effect on VSM; however, it appears that when all three estrogen receptors are co-expressed that the nuclear receptors play the more dominant role in slowing proliferation. At present, however, potential cross-talk between GPER and ERα or ERβ and signaling events downstream from GPER activation in VSMC is unknown. Potential effects of G-1 on downstream signaling events are also unknown.

Our flow cytometry results indicate that G-1 increases the number of cells in the G1-phase, and hinders cell cycle transition into the S phase and G2/M phase. Moreover, G2/M phase-specific cyclin B1 was strongly down-regulated by G-1. Cell-cycle progression from the G1-phase of the cell cycle to S phase entry is tightly regulated by cyclin-dependent kinases and their cyclin-regulatory subunits. Because cyclin B1 is essential for G2/M phase transition entry into mitosis, our finding of G-1-induced down-regulation of cyclin B1 is consistent with an anti-proliferative effect of G-1 on CASMCs. In addition to cyclin B1, cyclin D1 gene is frequently overexpressed in VSMC under growth factor stimulation, and its down-regulation has been proposed to be associated with G1-phase arrest of cell growth [37], [38]. Unexpectedly, we found that expression of G1-S phase specific cyclin D1 was up-regulated by G-1 in VSMCs. Obviously, cyclin D1 was not involved in G-1-induced inhibition of cell cycle progression from G1- into S-phase. This finding seems to differ from a previous report where 17β-estradiol suppressed PDGF-stimulated progression from G1- to S-phase in human aortic artery smooth muscle proliferation, possibly by inhibiting PDGF-induced phosphorylation of retinoblastoma protein (pRb) or by reducing cyclin D1 expression [10]. Therefore, GPER may function differently from ERα and ERβ: GPER does not mediate inhibition of cyclin D1 expression nor pRb phosphorylation in VSMC. Although G-1-induced up-regulation of cyclin D1 is unexpected, it is not surprising as studies have shown that cell differentiation can be promoted by up-regulation of cyclin D1 and p21 [28].

The re-differentiation process of synthetic VSMC is coupled to withdrawal from the proliferation cell cycle, and we examined how G-1 affects smooth muscle cell differentiation marker protein expression in both porcine and human CASMCs. Differentiation induced by SMDS [39], [40] changed cellular morphology from slender stellate cells to enlarged rectangular shaped cells, and VSMC α-actin content was clearly increased, as predicted. However, G-1 exerted an even stronger effect than SMDS on cell morphology and α-actin expression in both human and porcine CASMCs. Collectively, these data certainly demonstrate that GPER activation induces VSMC differentiation phenotype and repression of cell cycle progression. The mechanism of how this repression is coupled to re-differentiation is beyond the scope of the present study, but evidence suggests that increased p21 and cyclin D1 expression is correlated with differentiation of various cell types [29], [41], [42]. We found increased expression of p21 and cyclin D1 as early as 6 hours after exposure to G-1, and this effect persisted up to 72 hours – an effect which correlated with increased expression of VSMC α-actin and SM22 α at the first and second day of CASMCs exposure to G-1. Taken together, these findings suggest that G-1-induced CASMC differentiation is related to greater expression of p21 and cyclin D1.

GPER has been shown to modulate ERK1/2 and Akt activity, which is dependent upon trans-activation of the epidermal growth factor (EGF) receptor via release of heparin-bound EGF (HB-EGF) in breast cancer cells [25]. We found that although phosphorylation levels of ERK1/2 and Akt in human and porcine CASMCs fluctuated somewhat over time, the overall effect of G-1 was to decrease phosphorylation. Pretreating CASMCs with G-1 led to a significant concentration-dependent decrease in serum-stimulated ERK1/2 and Akt phosphorylation. Apparently, GPER exerts a negative effect on ERK and Akt signaling in the presence of mitogens in CASMCs. This finding is consistent with studies by Filardo et al. [26] where GPER activation stimulated adenylyl cyclase activity in breast cancer cells and suppressed EGF-induced ERK1/2 activity. Whether cAMP signaling is involved in the G-1 effect on ERK1/2 activity in the CASMCs is an ongoing investigation in our laboratory. The present study provides new evidence that GPER plays an important role in regulating coronary artery smooth muscle growth, and promotes re-differentiation and a contractile phenotype in these cells. Furthermore, we propose GPER as a novel therapeutic target to prevent coronary artery dysfunction.
