# Correction: Activation of GPER Induces Differentiation and Inhibition of Coronary Artery Smooth Muscle Cell Proliferation

**DOI:** 10.1371/annotation/ce509358-9f5c-4a8c-9b0d-80b4be3f4fc1

**Published:** 2013-07-10

**Authors:** Fen Li, Xuan Yu, Claudia K. Szynkarski, Cong Meng, Beiyan Zhou, Rola Barhoumi, Richard E. White, Cristine L. Heaps, John N. Stallone, Guichun Han

The images for Figures 4 and 5 were reversed. The legends of these figures are correct. 

